# Li-ion battery cooling system integrates in nano-fluid environment

**DOI:** 10.1007/s13204-016-0539-6

**Published:** 2016-10-19

**Authors:** Lien Tran, Jorge Lopez, Jesus Lopez, Altovely Uriostegui, Avery Barrera, Nathanial Wiggins

**Affiliations:** 1 University of Houston, Houston, USA; 2 San Jacinto College, Pasadena, USA

**Keywords:** Aerogel, Heat conduction, Heat extraction, Micro-fluid, Nanoparticle, Nanotechnology

## Abstract

In this design challenge by the Texas Space Grant Consortium, the researchers design a cooling system for a lithium-ion battery. Lithium-ion batteries are an effective and reliable source of energy for small, portable devices. However, similar to other existing sources of energy, there is always a problem with overheating. The objective is to design a cooling system for lithium-ion batteries that will work in a zero gravity environment for orbital and interplanetary space systems. The system is to serve as a backup battery and a signal booster that can be incorporated into a spacesuit. The design must be able to effectively cool the batteries without the use of an atmosphere to carry away heat but also be a lightweight and reliable design. The design incorporates carbon nanotubes suspended in distilled water creating a nano-fluid environment. This design must include a failsafe in the event of thermal runaway, a problem common to lithium-ion batteries. This failsafe will completely shut off the system if the batteries reach a certain temperature. A cooling system that incorporates nano-fluids will achieve a lightweight and efficient way of cooling batteries.

## Introduction

In the early 1970s, the first non-rechargeable/disposable lithium-ion batteries became commercially available. Today, rechargeable lithium-ion batteries are being used as a power source for many portable devices and in consumer electronics. In space, lithium-ion batteries provide power to the astronaut’s spacesuit, portable electronic devices, and satellites. A few benefits of lithium-ion batteries are as follows: it is the lightest of all the elemental metals that have a low maintenance battery, possesses the greatest electrochemical potential, and provides the largest energy density for weight. By ensuring certain precautions are met when charging and discharging, lithium-ion batteries prove to be safe. While there are several benefits of lithium-ion, a disadvantage in the continuous charging and discharging of these batteries is overheating (Baloch et al. 2010). The overheating can lead to thermal runaway, which causes the device to burst into flames and combust, resulting in possible injury to personnel or equipment (Wang et al. 2012). Therefore, for this experiment, the team is building a cooling system with the incorporation of nanotechnologies consisting of nano-fluid and aerogel. Carbon nanotubes are used as nanoparticles that mix with distilled water to form nano-fluid. Nanotubes are known to provide great surface areas making heat extraction more efficient. It is small and lightweight which yield size and weight flexibility of the model (Kostagiannakopoulou et al. 2016). Similarly, an aerogel pad is extremely lightweight, as its structure mostly consists of the air. It serves as a thermal insulator in the model to prevent environmental components affecting the system (Lizeng et al. 2015).

## Objective

Cooling systems play a significant role in keeping the battery cool by extracting the heat and keeping everything at a balanced temperature. The researcher’s objective is to design a cooling system for both cylindrical and flat lithium-ion batteries that will work in a microgravity environment. Although both designs will be used in space, the flat battery is being designed for astronaut spacesuits with safe and lightweight characteristics in mind. In order for the lithium-ion batteries to perform at its most favorable capacity, the researchers have devised a better cooling method while maintaining optimal temperatures. The idea behind this cooling method lies in the cooling system transferring the heat to external heat exchangers. These heat exchangers then radiate the heat out into space causing high temperatures to affect the lithium-ion battery drastically (Baloch et al. 2010). Thermal abuse may be a result due to external and/or internal factors. With thermal abuse, there will be an increase in rate of performance, but consequently, it will also degrade the lithium-ion battery’s life (Tables 1, 2).

**Table 1:** Mean values of both fluids are 83.850 and 80.850

	Nano-fluid exp.	DI water exp.
Mean	83.850	80.850
SD	1.733	0.883
SEM	0.548	0.279
N	10	10

Data show nano-fluid experiment that is more efficient in heat extraction compared to distilled water alone

**Table 2:** Two tailed *p* value, confidence interval, and intermediate value are shown above, indicating that the expected value is close to be identical with the theoretical value

Two tailed *p* value	Confidence interval	Intermediate value
*p* = 0.0001	Mean = −3.0000	*t* = 4.8786
Extremely significant	95 % Confidence (−4.2919 to −1.7080)	Standard error of difference = 0.615

The United States Department of Energy’s Office of Energy Efficiency and Renewable Energy has set the temperatures between 14 °F (−10 °C) and 86 °F (30 °C) to be the optimal range for lithium-ion battery performance. External temperatures may cause short-circuiting of the battery because of Joule heating (I2R), the cell then begins to produce heat by internal chemical reactions. Battery temperatures exceeding 86 °F (30 °C) begin to accelerate the rate of performance, which may result in thermal runaway. Thermal runaway occurs when an exothermic reaction goes out of control, that is, the reaction rate increases due to increasing temperature resulting both temperature and rate. This phenomenon will cause the lithium-ion battery to possibly combust and catch on fire. In addition, NASA has a set of battery guidelines that exist to prevent the propagation of thermal runaway. Therefore, with NASA’s battery guidelines in mind, the team’s goal is to integrate the use of nanotubes within the cooling system to enhance battery performance.

## Methodology

The main purpose is to power a space suit through a backup battery and signal booster with an incorporated cooling system. To increase the surface area for heat extraction, nanotubes are integrated into the cooling system to surround the batteries, while it is suspended in distilled water (Nemilentsau and Rotkin 2012). Due to its high thermal conductivity at around 3500 W/(m K), carbon nanotubes have been considered as one of the most efficient heat transfer additives available. The porous matrix of nanotubes aid in even distribution of heat discharge, which promotes heat transfer and cools the system (Li et al. 2013).

**Fig. 1:**
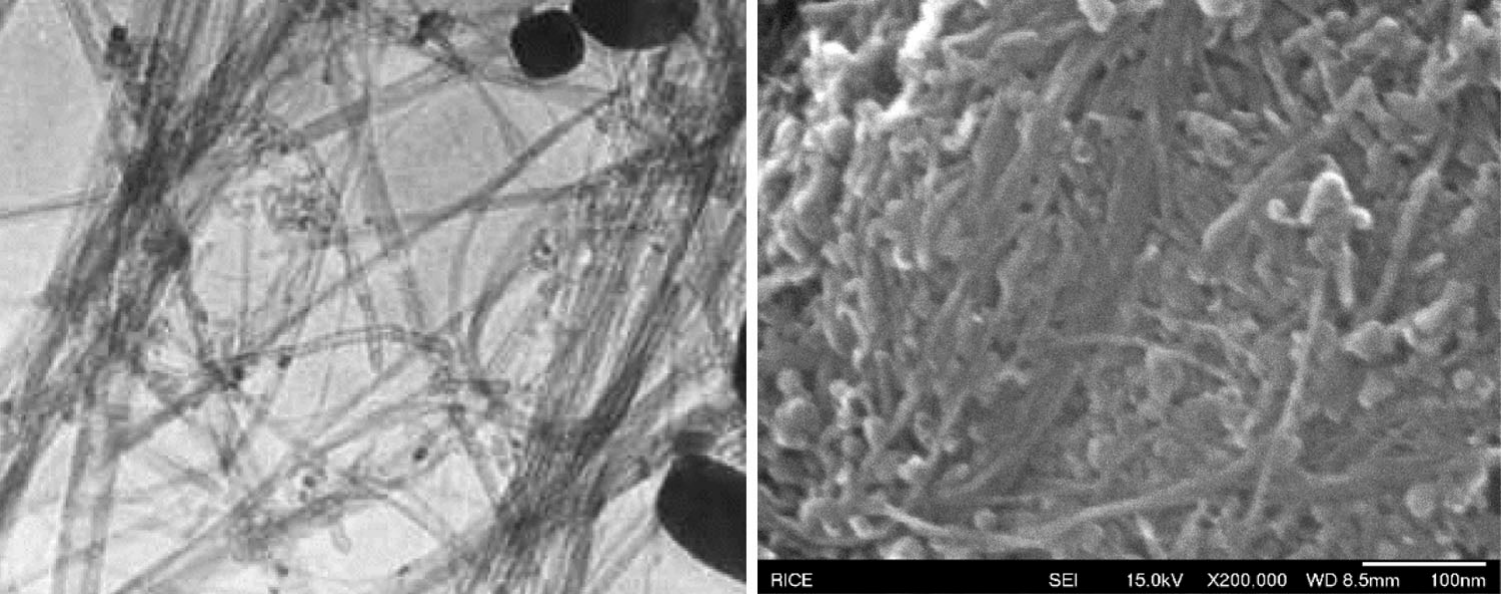
*Left* Image shows the SWCNTs (single-walled carbon nanotube) before being suspended in water (Foley 2016).* Right* SEM image shows the attempted-drying SWCNTs after suspended in distilled water

**Fig. 2:**
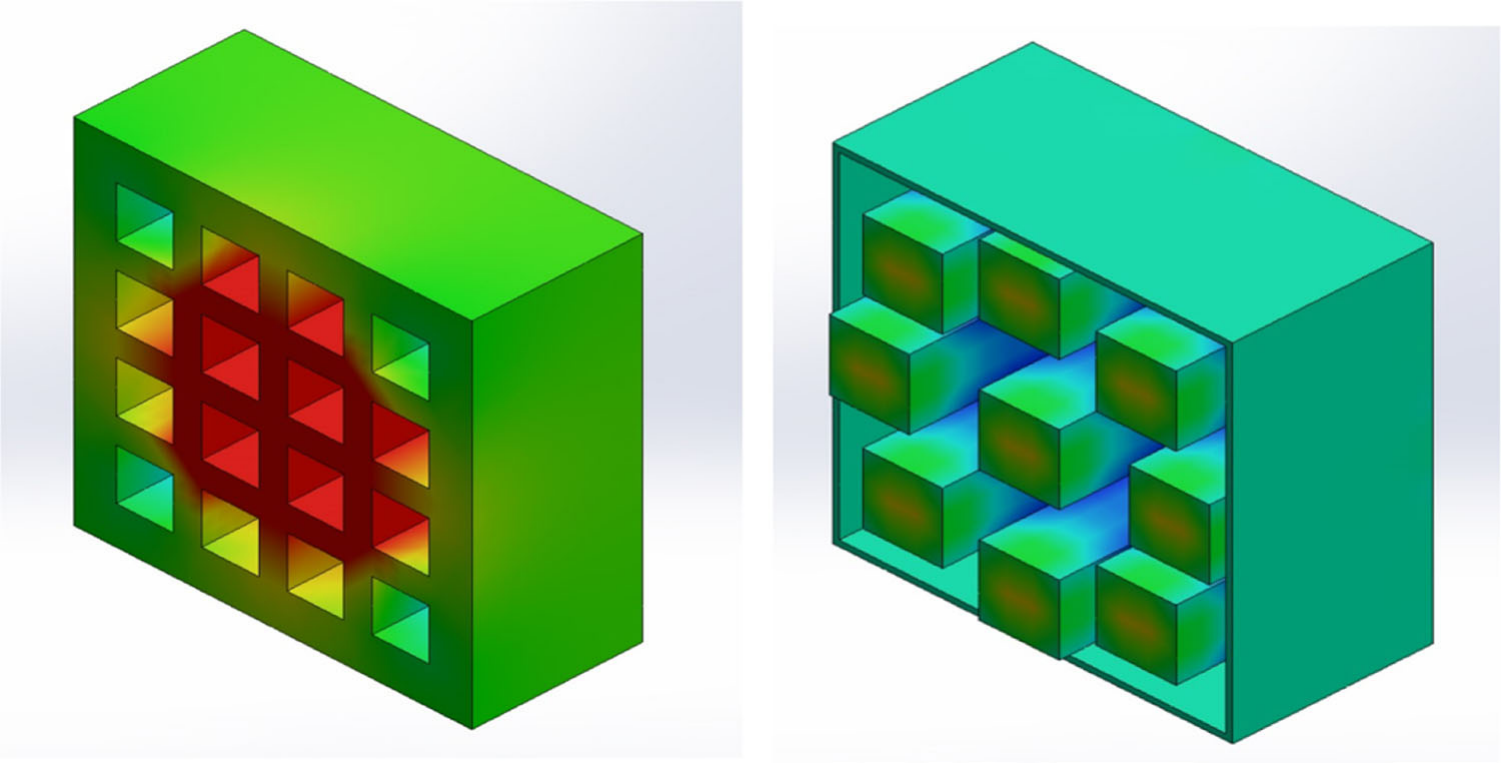
*Left* Original design, * right* new design. The original design shows greater heat accumulation in the center cells. By changing the orientation and layout of the cells (*right image*), heat is evenly distributed. Component design and thermal simulation

**Fig. 3:**
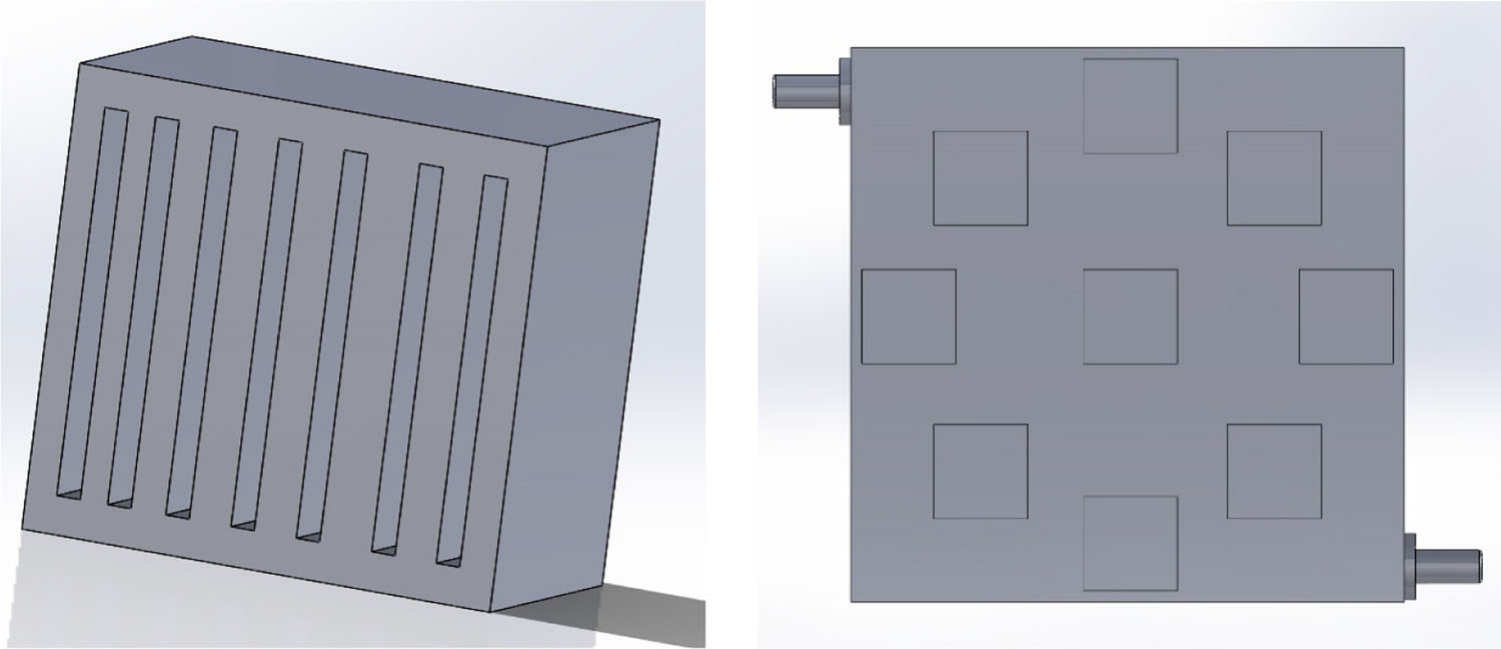
Versatility of the designs is shown. *Left image* is designed for flat cell batteries, while *right image* is designed for cylindrical cell batteries. Models created in Solidworks 2014

**Fig. 4:**
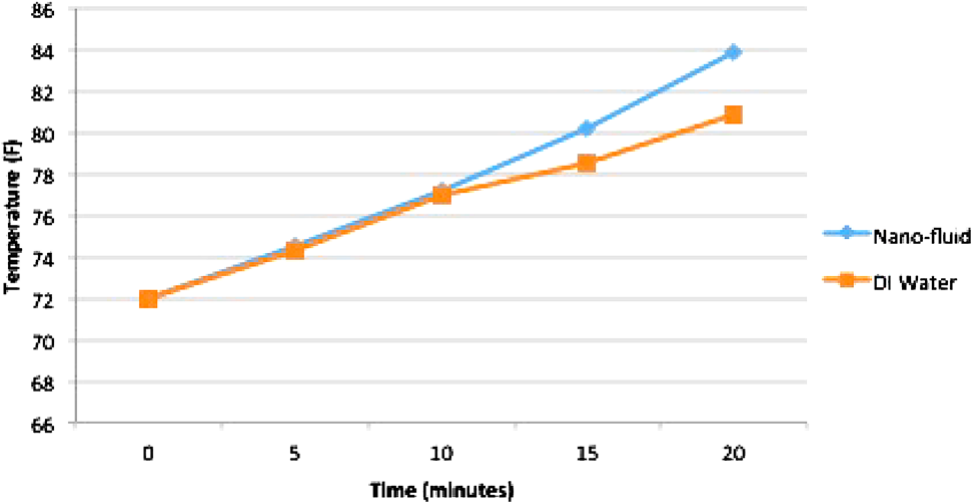
Nano-fluid requires 15 min to reach 80 °F. DI water takes 20 min to reach the same temperature proving the efficiency of nano-fluid

The nano-fluid will be pumped and circulated around the system—going through our battery-housing unit, as it cools the battery packs by heat conduction. The system is tested with two different fluids and two types of batteries—the nano-fluid with distilled water and lithium-ion batteries (flat) and cylindrical (3.7 V 3000 mA h). Due to its versatility, the design is expected to function with either batteries and is also anticipated to be used for other batteries systems. Sheets of aerogel surrounding the enclosure serve as the perfect insulator that will help negate external variables in a zero gravity environment. The aerogel prevents the external heat transfer to the environment by trapping the heat inside the model (Worsley et al. 2010). This enhances the effectiveness of the nano-fluid by lowering thermal conductivity. The nano-fluid is held in a small tank, where it will be pumped into the battery enclosure. Whilst in the enclosure, it will draw heat away from the batteries then get pumped into the radiator before repeating the cycle (Figs. 1, 2, 3, 4).

In addition, the incorporation of aerogel will isolate the system by preventing external and internal variables from affecting the system and preventing the spread of possible thermal meltdown. Aerogel is known to have a comparatively lower density than any solid with an extremely small microstructure, and it has the benefit of being extremely fire resistant (FAN 2015). Aerogel can withstand a high amount of heat (Hsieh et al. 2015). To monitor the temperature of the system, a temperature sensor, which is connected via Bluetooth to an Arduino board, is used. A notification is transmitted if the temperature rises to a critical point and at which it can be remotely shutdown if needed.

To provide evidence that the center battery received the most punishment, thermal tests were conducted. Evidence shows that the most heat is accumulated in the center battery which could cause thermal runway. By leaving some open space and not dedicating the entire area for holding batteries, the new design reduces possible occurrences of thermal runaway through efficient air cooling which greatly decreases the rate of failure.

## Experiments

For the experimentation, the researchers run tests to compare the effectiveness of nano-fluid to di-ionized water. A total of ten experiments were conducted in which submersible heaters heated both liquids from their initial temperatures of 72 °F. In each experiment, roughly 1 g of nanotubes were added into a gallon of di-ionized water. This yields a molar composition ratio of 99.96 % DI water and 0.04 % carbon nanotubes. Previous studies have shown that in presence of sodium dodecyl sulfate surfactant, thermal conductivity of nano-fluid is 0.75 W/(m K) with 0.05 vol % of nanotube used, which increases by 9.36 % compared to distilled water without nanotube. These data indicate higher volume of SWCNT contributes to higher thermal conductivity of the nano-fluid. High concentration of nanotube tends to promote agglomeration; however, agglomeration can be reduced to minimal using the sonication method to improve the stability of the nanoparticles (Sabiha et al. 2016).

## Results

The data points from all ten trial tests were averaged out to create a linear relationship between the changes in temperature over time. As shown in the table, the nano-fluid extracts heat at a faster rate than di-ionized water. The nano-fluid reached 80  F in 15 min, while di-ionized water took ~20 min to reach the same temperature. According to the statistical *t* test, there is a mean difference of about three degrees and the result is 95 % confident that in the given time interval, the nanotube solution would be between 1.71 and 4.29  F warmer than it would be if the de-ionized water was used. With the *p* value of 0.0001, our results are statistically significant.

## Conclusion

The group designs a cooling system for a cylindrical and flat battery and then examines results for best use to avoid overheating the battery and creating malfunctions (Loyselle et al. 2009). NASA’s guidelines are followed to produce an ideal, safe, and effective cooling system that minimizes the threat of thermal runaway by incorporating nano-fluid and aerogel into the system. The design will not be limited to battery size or quantity. The potential applications for the battery cooling systems include being able to run a system without worrying about it overheating not just for astronauts but for generally improving batteries in everyday uses, such as cell phones, cameras, and computers to name a few.

Future project plans may include, but are not limited to, performing further experiment of multiple nano-fluids with variation in concentration of the nanotubes, creating a system of more efficient batteries to control more than just small components, and reducing the cost of battery cooling system while maintaining or increasing its integrity. With this prototype, the researchers pursue further experimentation with different types of materials to improve the system efficiency.
